# Hemp seeds attenuate loperamide-induced constipation in mice

**DOI:** 10.3389/fmicb.2024.1353015

**Published:** 2024-04-04

**Authors:** Huang Hua, Wang Yongtong, Ding Xufeng, Li Fang, Gu Jing, Zeng Fumao, Jiang Jie, Ji Lijiang

**Affiliations:** ^1^Department of Anorectal Surgery, Changshu Hospital Affiliated to Nanjing University of Chinese Medicine, Changshu, China; ^2^School of Food Science and Resources, Nanchang University, Nanchang, China

**Keywords:** hemp seed, hemp seed extract, constipation, intestinal flora, short-chain fatty acids

## Abstract

Constipation is a common gastrointestinal disease that seriously affects human physical and mental health. Studies have reported that hemp seeds can improve constipation, however the specific mechanism is still unclear. This study investigates that hemp seed (HS) and its water-ethanol extract (HSE) attenuates loperamide-induced constipation in mice. The research results show that: the fecal water content and small intestinal transit rate of mice in the hemp seed group and hemp seed hydroalcoholic extract group were significantly increased compared with MC group, and the first red feces defecation time was significantly shortened; HS and HSE significantly influence serum levels of Gastrin (Gas), motilin (MTL), substance P (SP), and endothelin (ET), potentially mediating their effects on gastrointestinal motility. HS and HSE can improve colon inflammation in constipated mice with H&E staining. Compared with the model of constipation group, the content of short-chain fatty acids in the HS group and HSE group increased significantly. Gut microbiome studies have shown that the structure and abundance of intestinal flora are altered. HS and HSE changed the abundance of *Odoribacter, Bacteroide, Lactobacillus and Prevotella*. Together, these results suggest that HS have the potential to stimulate the proliferation of beneficial gut microbes and promote intestinal motility, thereby improving gut health and relieving symptoms of constipation.

## 1 Introduction

Constipation, a term used to describe a variety of symptoms, including hard stools, over-stress, frequent bowel movements, abdominal distension and abdominal pain, is one of the most common gastrointestinal disorders worldwide (Camilleri et al., 2017). According to epidemiological data, the global prevalence of constipation in adults is 18.9% (Salari et al., 2023). It is well known that the occurrence of constipation imposes a significant economic burden and serious psychological impact on the patients. It is estimated that the cost of purchasing laxatives exceeds $800 million per year in the United States (Wang et al., 2022). Chronic constipation can induce the accumulation of pathogenic bacteria in the colon and is associated with an increased risk of gastrointestinal disorders, such as irritable bowel syndrome and colorectal cancer (Heidelbaugh et al., 2015). Currently, laxatives are the first choice for constipation. However, prolonged use of these laxatives will cause some adverse effects, such as abdominal cramps, rashes, excessive flatulence and dizziness (Ding et al., 2016), more than 50% of patients are not completely satisfied with the treatment of constipation (Huang et al., 2022). Therefore, it is of practical significance to develop a simple and affordable method to improve constipation.

Hemp seeds (HS) are the dried ripe fruit of *Cannabis sativa* L. (Moraceae). Hemp seeds have been utilized both as a food and medicinal ingredient in traditional Chinese medicine for at least 3,000 years (Yan et al., 2015). They have the effects of preventing constipation, promoting cardiovascular health, regulating immunity, and treating skin and gastrointestinal disorders. Hemp seeds have been listed as a Chinese herbal medicine of affinal drug and diet by the National Health Commission of the People's Republic of China. Hemp seeds are rich in nutrients and natural active ingredients. As one of the most commonly used traditional Chinese medicine for the treatment of constipation, hemp seeds are rich in plant proteins, unsaturated fatty acids, vitamins and other nutrients, containing up to 28% of dietary fiber (Opyd et al., 2020). Modern pharmacology has confirmed that hemp seeds contain fatty oil, which can stimulate the intestinal mucosa to increase secretion, promote peristalsis and reduce water absorption in the large intestine, thereby exerting a laxative effect. In a population-based cohort study of 216 patients by Zheng et al., Chinese patent drug Dama Wan (literally Cannabis pills) prepared from hemp seeds and other raw materials was shown to have a reliable and safe efficacy in functional constipation at a dose of 7.5 g bid (Cheng et al., 2011). Huang L. S. et al. (2018) reported the efficacy of Maziren Wan (literally hemp seed pills) in stimulating intestinal mucosa, reducing water absorption in the intestine, softening feces, restoring gastrointestinal homeostasis to increase intestinal peristalsis and relieve constipation. A study by Cheng et al. concluded that hemp seeds improved colonic transit, increased the bowel movement frequency, and reduced the severity of constipation in patients with functional constipation (Zhong et al., 2019).

Currently, hemp seeds are primarily used in the form of hemp seed oil (HSE) or traditional Chinese decoction, which leads to a low utility of many beneficial ingredients in hemp seeds and production of waste residue. Moreover, there are few research reports on utilization of complete hemp seed to treat constipation. Therefore, we aimed to study the protective effect of HS and HSE in mice with loperamide-induced constipation.

## 2 Materials and methods

### 2.1 Materials and instruments

Hemp seeds were purchased from Nanjing Traditional Chinese Medicine Market. Loperamide hydrochloride were purchased from Yifeng Pharmacy in Nanchang. Gastrin (Gas), motilin (MTL), substance P (SP), acetylcholinesterase (AchE), endothelin (ET), vasoactive intestinal peptide (VIP) enzyme-linked immunosorbent assay (ELISA) kit had been purchased from Shanghai Yuanju Biotechnology Center. Hematoxylin and eosin (H&E) dye, paraformaldehyde (Wuhan Qian Baidu Biotechnology Co., Ltd.). Short-chain fatty acids (SCFAs) standards: acetic acid, propionic acid, butyric acid, isobutyric acid, valeric acid, and isovaleric acid standards were purchased from Shanghai Aladdin Biochemical Technology Co., Ltd.

5804R refrigerated centrifuge, 5424R refrigerated centrifuge [Ebende (Shanghai) International Trade Co., Ltd.]; SB-02 high-speed multifunctional crusher (Shanghai Puheng Information Technology Co., Ltd.); RT-6000 microplate reader {Leica DM500 upright microscope [Leica Microsystems (Shanghai) Trading Co., Ltd.]; Agilent 7890B gas chromatograph [Agilent Technologies (China) Co., Ltd.]}.

### 2.2 Experimental methods

#### 2.2.1 Preparation of feed supplemented with hemp seeds

Hemp seeds were added to the grinder and crushed into uniform particles for 3 min. Grind the maintenance feed for 3 min into powder. In every 90 g of maintenance feed, 10 g of crushed hemp seeds were evenly mixed to crush particles, then put into the mold for plasticity. The oven was heated at 60 degrees Celsius for 8 h to remove excess water and fix the shape, and 10% hemp seeds were made into added feed.

#### 2.2.2 Preparation of hemp seed extract

We accurately weighed 20.0 g of hemp seed, ground them, and added 200 mL distilled water according to the solvent ratio of 1:10, vortexed and mixed it, ultrasonic at 40°C for 1 h, centrifuged at 4,500 rpm/min for 5 min, and pour the supernatant into a clean vial. Ethanol solution (200 mL) was added 1:10 to the residue, vortexed and mixed, and the above steps were repeated. Water extraction and alcohol extraction were repeated three times, respectively, and the above extracts were combined. Rotary evaporation was carried out at the temperature of 50°C and the to a constant volume of 30 mL at a speed of 25 and extract concentrate (30 ml) was obtained and stored at −20°C for subsequent experiments.

#### 2.2.3 Animal experiment design

Forty specific pathogen-free (SPF) female BALB/c mice, 6–7 weeks old, weighing 16–19 g, were purchased from Spefford (Beijing) Biotechnology Co., Ltd. [license number: SCXK (Beijing) 2019–0010]; mouse maintenance feed was purchased from Jiangsu Synergy Biotechnology Co., Ltd. (Nanjing, China). The experimental animals were housed in a specific pathogen-free (SPF) facility, with unrestricted access to food and water, with a 12-h light-dark cycle, and the growth environment temperature was 23°C ± 2°C. After adaptive feeding for 3 days, the mice were randomly divided into four groups (*n* = 10): blank control group (CN: normal saline + maintenance feed), model of constipation group (MC: loperamide 10 mg/kg + maintenance feed), hemp seed group (HS: normal saline + 10% hemp seed powder added to feed), hemp seed extract group (HSE: hemp seed extract + maintenance feed). During the entire experiment, mice in the CN group were fed standard mouse chow without any treatment. After the adaptation period, mice in MC, HS, and HSE groups were intragastrically administered loperamide 10 mg/kg at 9:00 a.m. every day for 2 weeks (Huang et al., 2022). One week after modeling, the HSE group was intragastrically administered with the test substance (0.2 mL). The Hemp seed group were given free access to the maintenance feed containing with 10% hemp seed supplemented. When the number of defecation pellets of the mice in the constipation group is significantly different from the number of defecation pellets of the mice in the blank control group, it means that the constipation model has been successfully established. During the experiment, the body weight and food intake of the mice were recorded twice a week; the physiological and fecal status of the mice were observed every day.

### 2.3 Moisture content of feces

After the constipation model was successfully established, mouse feces were collected every day and photos were taken to record the appearance of the feces. Each group of mice was placed in a clean cage, deprived of food and water, and allowed to defecate freely for 30 min. Fresh feces were collected in a 1.5 ml dry centrifuge tube, and the resolved samples were weighed using an electronic balance. All fecal samples were dried in an electric constant-temperature drying oven at 60°C until the samples had a constant weight. The formula for calculating feces water content is as follows:

Moisture content of feces (%) = (feces weight before drying—feces weight after drying)/feces weight before drying ^*^ 100

### 2.4 Defecation experiment

Preparation of red ink: a solution of 6 g phenol red in 100 ml 1% carboxymethylcellulose, which was stirred continuously and maintained at 37°C (Crowe and Kinsey, 2017).

This experiment was conducted after the 21st day, and all mice were fasted and water-free for 12 h. After the fasting, 0.2 ml of red ink was administered into the stomach and each mouse was individually transferred to a clean empty cage. The time when each mouse first discharged red stool was recorded.

### 2.5 Small intestinal transit rate

This experiment was conducted after the 22nd day, and all mice were fasted and water-free for 12 h. After fasting, 0.2 ml of red ink was administered into the stomach, and the eyes were anesthetized for 10 min to collect blood and then killed by neck dissection. Measure the distance traveled by the red ink and the total length of the small intestine. The gastrointestinal transit rate is calculated according to the formula:

Gastrointestinal transit rate (%) = red ink advancement distance/total length of small intestine^*^100

### 2.6 Distal colon histopathology

Colon tissue was fixed with 4% paraformaldehyde, embedded in paraffin, and cut into 4 μm thick sections. Fixed sections were dehydrocarbonated with xylene, hydrated with graded ethanol, and rinsed with distilled water. Hematoxylin and eosin (H&E) staining kit was used to detect morphological changes in the colon and ileum.

### 2.7 Serum biochemical analysis

After the intestinal transport experiment, the mice were anesthetized with isoflurane, and the eyeballs were enucleated and blood was collected. All blood samples were centrifuged at 4°C, 3,500 rpm (5804R desktop refrigerated centrifuge) for 15 min, and serum samples were obtained and stored in a −80°C ultra-low temperature refrigerator. According to the instructions of the enzyme-linked immunosorbent assay (ELISA) kit, the gastric motility protein (MTL), gastrin (Gas), endothelin (ET), substance P (SP), acetylcholinesterase (AChE), and blood vessels in the serum were measured. Viable intestinal peptide (VIP) levels.

### 2.8 Determination of short-chain fatty acid content

Gas chromatography was used to determine the contents of acetic acid, propionic acid, butyric acid, isobutyric acid, valeric acid and isovaleric acid. Mouse feces were collected, quickly frozen in liquid nitrogen, and placed in an 80°C freezer for testing. Weigh 200 mg of feces into a 1.5 ml centrifuge tube, add 1 ml of ultrapure water, add 10 μl of concentrated hydrochloric acid to acidify, homogenize for 3 min, let stand for 20 min after homogenization, centrifuge at 10,000 rpm for 10 min in a high-speed centrifuge at 4°C, and remove the supernatant The liquid is transferred to the injection bottle for testing.

### 2.9 Intestinal microbial 16s rRNA sequencing

Before the end of the experiment, the sterile feces of each mouse were collected, quickly frozen in liquid nitrogen, and then stored in an −80°C freezer for later use. Mouse fecal samples were taken out. Magnetic Soil and Stool DNA Kit (TianGen) was used to extract genomic DNA from the samples. The purity and concentration of DNA were detected by 1% agarose gel electrophoresis. Appropriate sample DNA was placed in a centrifuge tube and diluted to 1 ng/μL with sterile water. Then the PCR products were obtained and purified. The Library was constructed using NEB Next? Ultra™ II FS DNA PCR-free Library Prep Kit (New England Biolabs). The constructed library was quantified by Qubit and Q-PCR. Enable NovaSeq6000 to perform PE 250 on-machine sequencing. According to Barcode sequence and PCR amplification primer sequence, the sample data were separated from the disembarkation data. Qiime2 is used to analyze the raw data obtained and generate a graph of the analysis results on the Yun Tutu platform.

### 2.10 Data analysis

SPSS 13.0 statistical software was used for analysis, and GraphPad Prism 9.0 software was used for graphing. Data were expressed as Mean ± standard error of the mean (SEM). One-way analysis of variance was used for comparison between groups. P<0.05 is considered as statistically significant.

## 3 Results

### 3.1 Effects of hemp seeds on physiological parameters in mice with constipation

**Figure 1:**
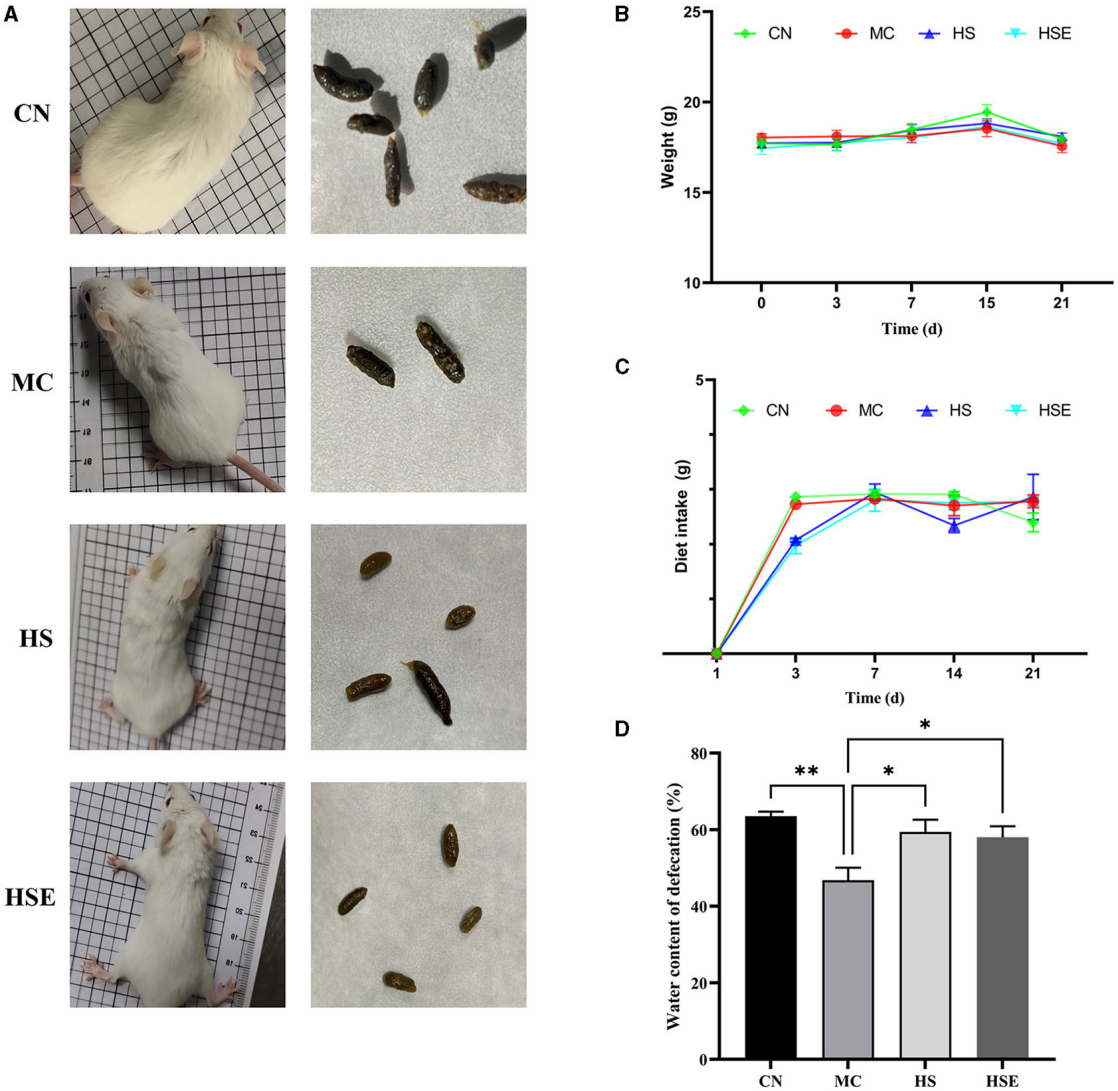
Effects of hemp seeds on the growth parameters and moisture content of feces in mice with constipation **(A)** Physiological status and feces of mice **(B)** Weight variation of mice **(C)** Food consumption of mice, and **(D)** Moisture content of feces. Results are provided as mean ± SEM (*n* = 4–6) ^*^P < 0.05, and ^**^P < 0.01 compared to constipation group.

The status and fecal morphology of the mice are shown in Figure 1A. Mice in the CN group appeared to be behaviorally active, with shiny hair and normal fecal pellets in size with a smooth and moist surface. In contrast, mice in the MC group were found to be less behaviorally active by curling themselves up, with fluffy hair and dark hard stools with a rough dry surface after induced constipation. After 2 weeks of treatment with HS and HSE, improvement in physiological state was observed in both HS and HSE groups. Mice in the HS group appeared to be behaviorally active, with fecal pellets that were smooth and soft, while mice in the HSE group were in a good mental state, with smaller fecal pellets that were smooth on surface and less hard. As shown in Figures 1B, C, there was no significant difference in weight variation and food consumption across the four groups. As shown in Figure 1D, there was a significant difference in water content of feces between the CN group and the MC group, that is, a significant increase in moisture content of feces was observed for mice in the HS and HSE groups after treatment with HS and HSE, respectively (*P* < 0.05).

### 3.2 Effects of hemp seeds on intestinal tract

**Figure 2:**
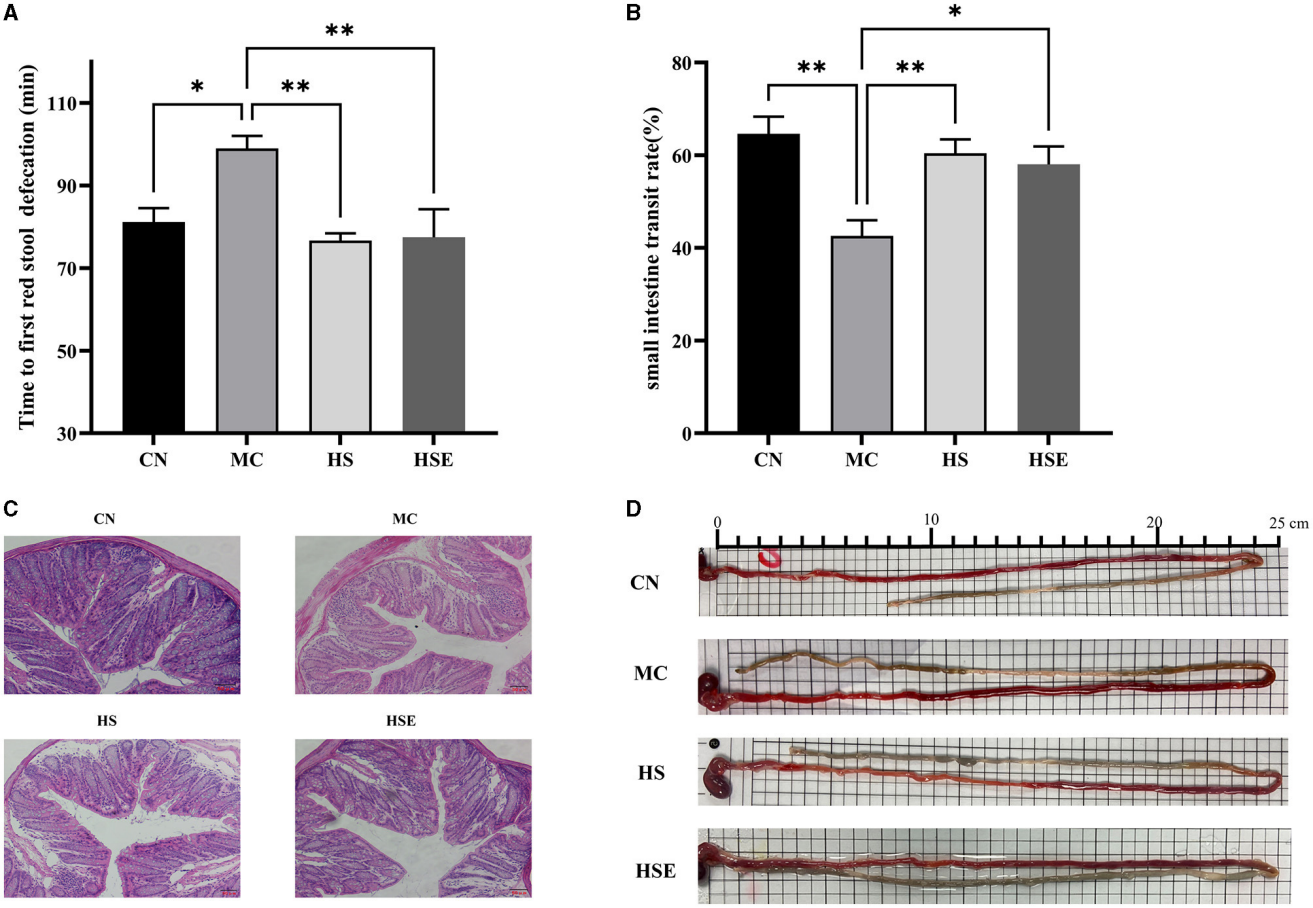
Effects of hemp seeds treatment on intestinal transit and protective effects on colonic tissue in mice with constipation **(A)** Time to discharge of the first red fecal pellet **(B)** Small intestinal transit rate **(C)** HE staining in the colon of mice, and **(D)** Image of assay on small intestinal transit rate (each cell in the picture is 0.5 cm long). Results are provided as mean ± SEM (*n* = 5–6) *P < 0.05 and **P < 0.01 compared with constipation group.

The time to first bowel movement and small intestinal transit rates of the mice are provided in Figures 2A, B, D. After induced constipation, the time to first bowel movement was significantly prolonged in the MC group compared with the CN group (*P* < 0.05), while the small intestinal transit rate was significantly decreased (*P* < 0.01), conforming to the characteristics of constipation in mice. After treatment with HS and HSE, the time to first bowel movement was significantly reduced in the HS and HSE groups (*P* < 0.01). In terms of improvement of small intestinal transit rate, the HS group showed an extremely significant change (*P* < 0.01) whereas the HSE group showed a significant change (*P* < 0.05). The experimental results showed that HS was superior to HSE in terms of the effects of reducing the time to bowel movement and promoting small intestinal transit. To further investigate the protective effects of HS and HSE on the intestinal tract in mice with constipation, we observed the histomorphological changes of the distal colon by histological staining. As shown in Figure 2C, increased infiltration of inflammatory cells, shorter crypts, fewer goblet cells and greater muscularis-crypt distance were observed in the colon of the MC group compared with the CN group. After treatment with HS and HSE, decreased inflammatory cell infiltration and increased goblet cells were noticed, indicating that constipation induced colitis in the mice and HS/HSE could relieve the inflammatory reaction caused by constipation.

### 3.3 Effects of hemp seeds on serum levels of neurotransmitters relating to gastrointestinal regulation

**Figure 3:**
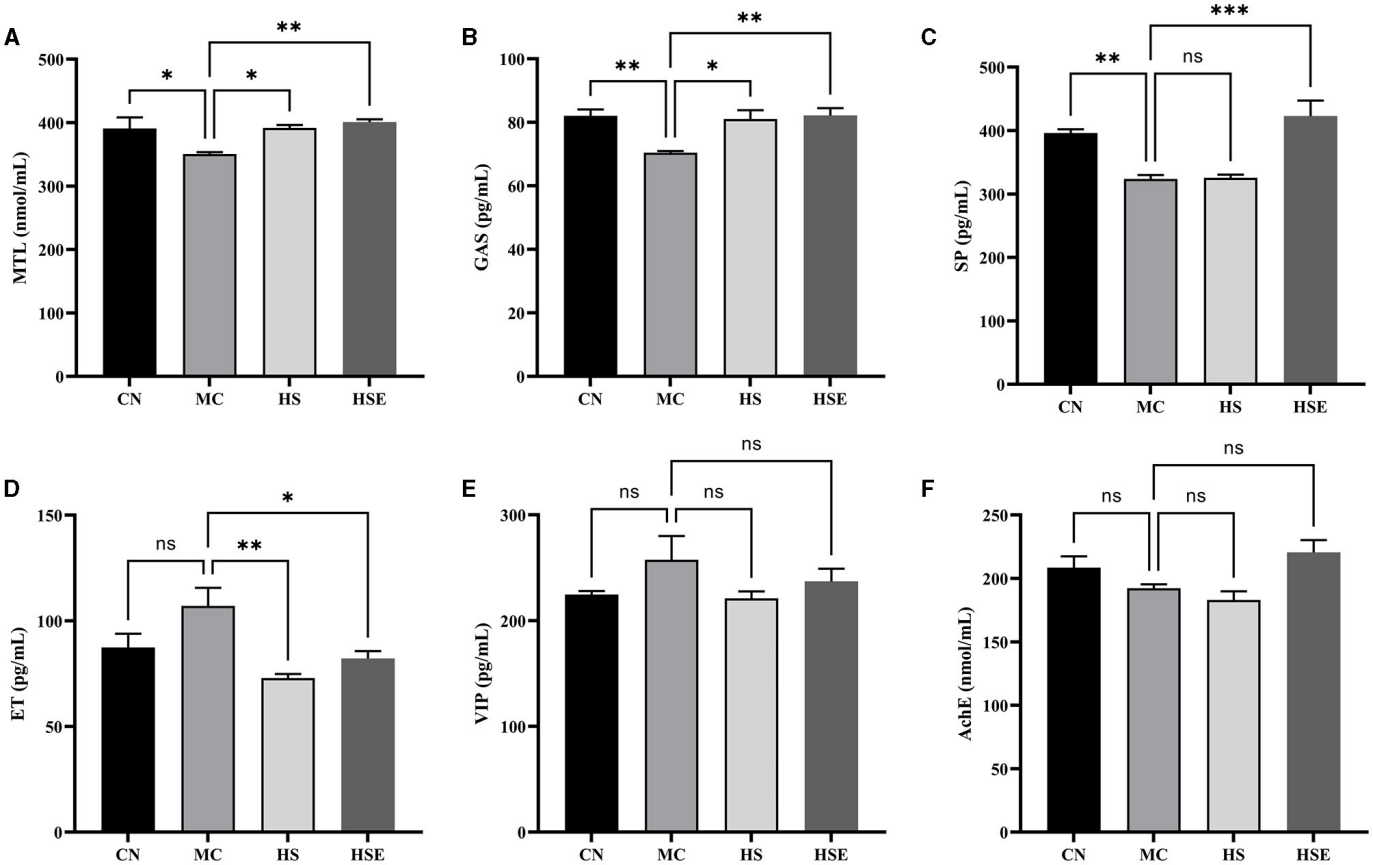
Effects of hemp seeds treatment on serum levels of neurotransmitters relating to gastrointestinal regulation in mice with constipation. **(A–F)** represents MTL, GAS, SP, ET, VIP, and AchE, respectively. Results are provided as mean ± SEM (*n* = 4) *P < 0.05, **P < 0.01, and ***P < 0.001 compared with constipation group.

To further evaluate the effects of HS and HSE on constipation, serum parameters were measured. As shown in Figure 3, the levels of MTL, GAS and SP in the MC group were significantly lower than those in the CN group (^*^P < 0.05 for MTL, ^*^P < 0.05 for GAS, and ^**^P < 0.01 for SP), while the levels of ET and VIP increased but were not significantly different from those in the CN group, and the level of AchE decreased but was not significantly different from that in the CN group. After treatment with HS, the levels of MTL and GAS were significantly increased (^*^P < 0.05), and the level of ET was significantly decreased (^**^P < 0.01), but there were no significant differences in the levels of SP, VIP and AchE. In contrast, HSE-treated mice were observed with significant increases in the levels of MTL, GAS, and SP (^**^P < 0.01 for MTL, ^**^P < 0.01 for GAS, and ^***^P < 0.001 for SP), and a significant decrease in the level of ET (^*^P < 0.05), whereas there were no significant differences in the levels of VIP and AchE.

### 3.4 Effects of hemp seeds on short-chain fatty acids

**Figure 4:**
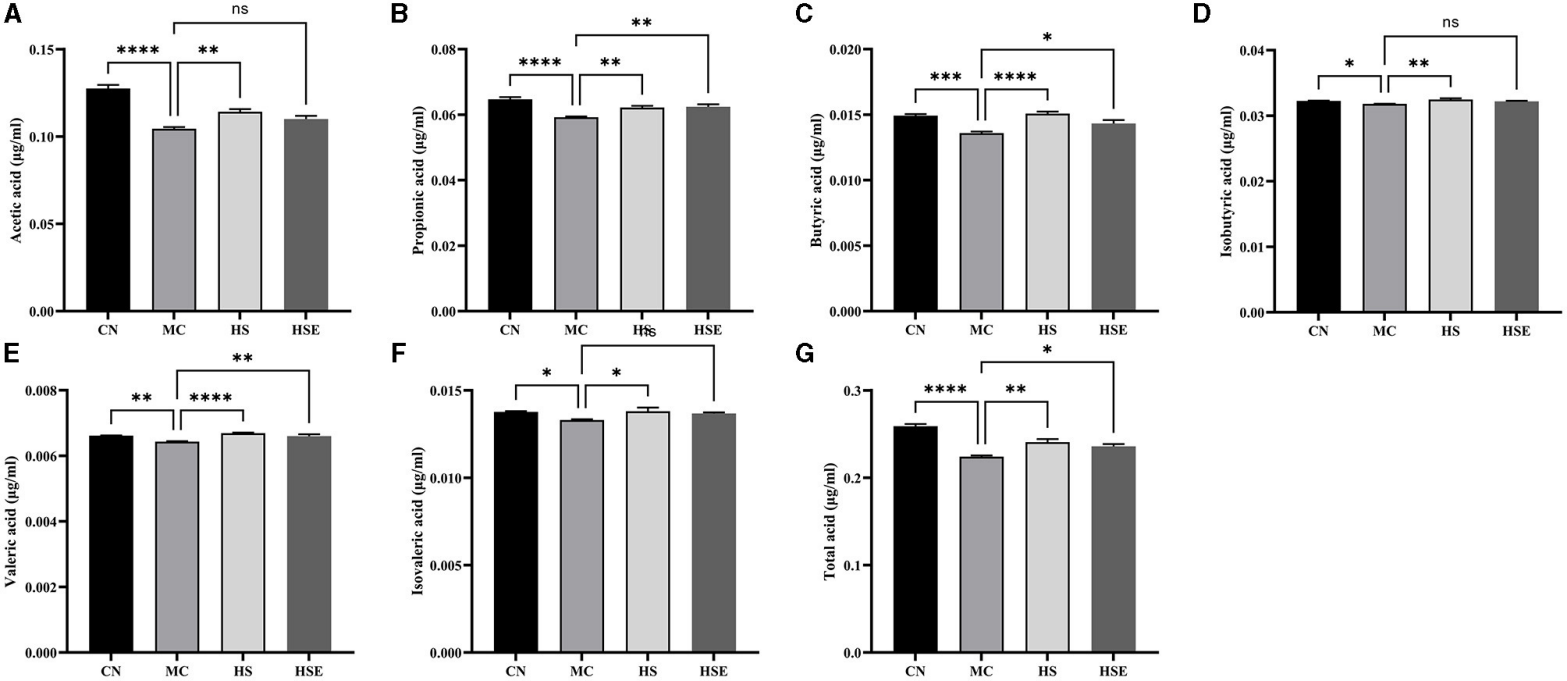
Effects of hemp seeds treatment on short-chain fatty acids (SCFAs) in the mice with constipation. **(A–G)** represents acetic acid, propionic acid, butyric acid, isobutyric acid, valeric acid, isovaleric acid and total acids, respectively. Results are provided as mean ± SEM (*n* = 6) *P < 0.05, **P < 0.01, ***P < 0.001, and ****P < 0.0001 compared with constipation group.

SCFAs are the main products from the fermentation of indigestible carbohydrates by symbiotic bacteria. In addition to the effects of inhibiting the growth of pathogenic microorganisms and increasing the absorption of certain nutrients, SCFAs were also reported to regulate intestinal neurons and affect gastrointestinal motility. The fecal concentrations of SCFAs in this study are provided in Figure 4. The fecal concentrations of acetic acid, propionic acid, butyric acid, isobutyric acid, valeric acid, isovaleric acid and total acids were significantly lower in the MC group than in the CN group. After HS treatment, the HS group was observed with a significant increase in the fecal concentrations of the six short-chain fatty acids, while the HSE group was noticed with a significant increase in the fecal concentrations of propionic acid, butyric acid, valeric acid and total acids compared with the MC group, without no significant change in the fecal concentrations of acetic acid, isobutyric acid and isovaleric acid. The results showed that HS/HOS treatment led to a change in the content of SCFAs, which was possibly the protective mechanism of HS and HSE on the mice with constipation.

### 3.5 Effects of hemp seeds on the composition of intestinal microorganisms

**Figure 5:**
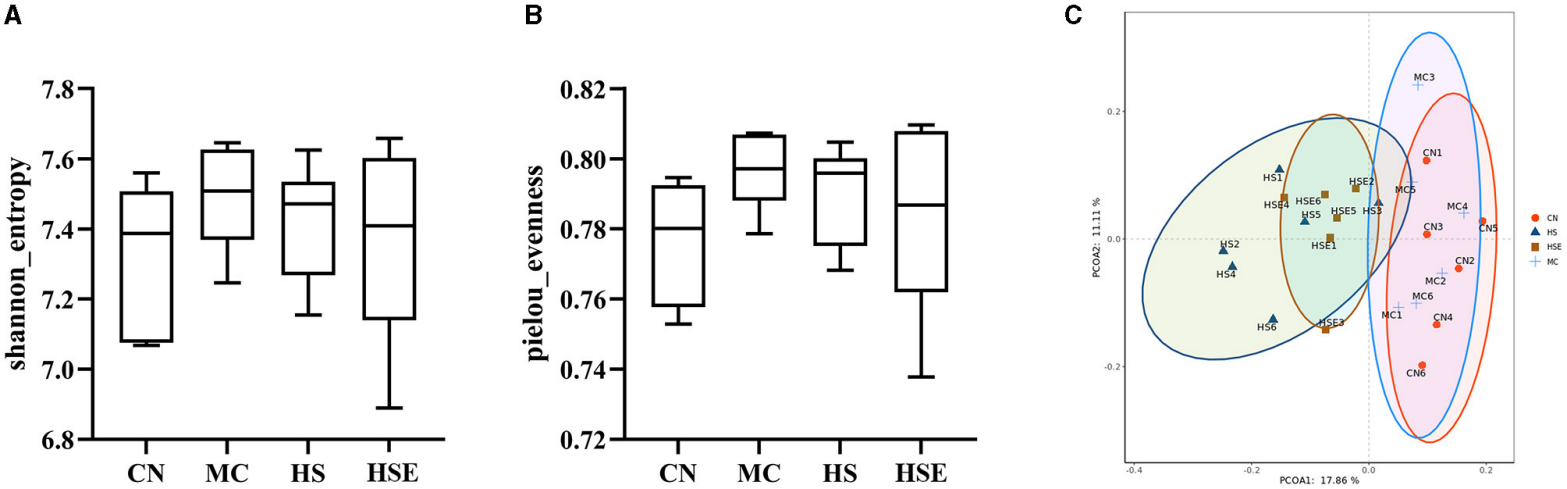
Review on the composition and diversity of intestinal microbiota in mice treated with hemp seeds for constipation. **(A)** Shannon Index, **(B)** Evenness Index, and **(C)** Plot of Principal Coordinate Analysis (PCoA) Score. Data are provided as mean ± SEM (*n* = 6).

To assess the effects of hemp seeds on the microbiota in intestinal feces, we performed metagenomic analysis of 16SrRNA gene sequences to examine changes in intestinal microorganisms after prophylaxis with hemp seeds (HS, HSE) and to characterize the effects on intestinal microbiota in mice after loperamide treatment. Alpha diversity (encompassing multiple indexes) is used as a measure of microbiota diversity (Cao et al., 2021). Totally 24 samples from four groups were evaluated. After sequence optimization, a total of 22,711,313 readable sequences were generated, with an average of 112,972 readable sequences per group. The results and the calculated α-diversity indexes of microbiota are shown in Figures 5A, B. At the OUT level, after Loperamide induced constipation, the Shannon and pielou_evenness indexes of intestinal microbiota in the MC group were higher than those in other groups, but there was no significant difference between groups as compared, indicating that HS and HSE treatment had no significant effect on the α-diversity of microbiota. As shown by the results of β-diversity (as measured by the Bray-Curtis distance) in Figure 5C, a trend toward segregation was observed with mice in the CN and MC group, whereas the HS and HSE groups were largely segregated from the MC group.

**Figure 6:**
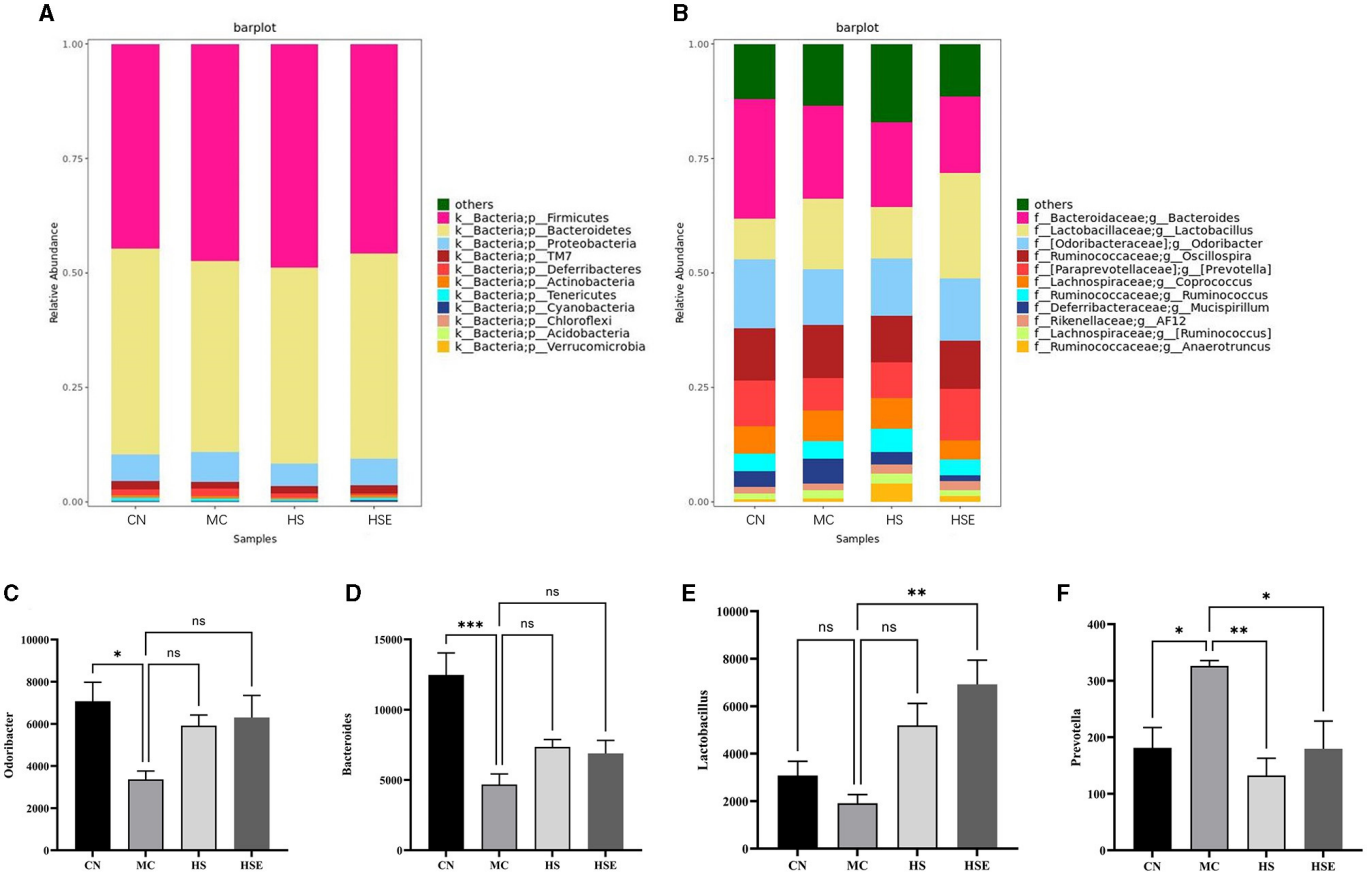
review on relative abundance of intestinal microbiota in mice treated with hemp seeds for constipation- composition of the microbiota. **(A)** phylum and **(B)** genus levels, **(C)** Relative abundance of *Odoribacter*, **(D)** Relative abundance of *Bacteroides*, **(E)** Relative abundance of *Lactobacillus*, **(F)** Relative abundance of *Prevotella*. Data are provided as mean ± SEM ^*^P < 0.05, ^**^P < 0.01, and ^***^P < 0.001 compared with constipation group.

Taxonomically, Figure 6A presents the distribution of fecal flora at the phylum level. Although a total of 11 phyla were identified in the feces from all mice, the fecal flora was mostly composed of *Firmicutes/Bacteroides*. Therefore, we further conducted a discussion on changes in the proportion of the two phyla. Compared with the CN group, the proportion of *Bacteroides* was decreased and that of *Firmicute* was increased in the MC group, and the *Firmicutes/Bacteroides* ratio was 1.14 in the MC group, being higher than the ratio of 0.99 in the CN group, without a significant difference. After treatment with HS/HSE, the proportion of *Firmicutes* increased in the HS group, and interestingly, the number of *Bacteroides* also showed a trend toward increasing in the HS group compared with the MC group, but the *Firmicutes/Bacteroides* ratio in the HS group was almost equal to that in the MC group. On the other hand, the proportion of firmicutes decreased but that of *Bacteroides* increased in the HSE group compared with the MC group, and therefore the *Firmicutes/Bacteroides* ratio in the HSE group was lower than that in the MC group, but there was not a significant difference, which was consistent with the study findings (Lin et al., 2022). At the genus level, as shown in Figures 6B–F, the proportions of *Bacteroides* and *Odoribacter* were significantly lower in the MC group than in the CN group. Interestingly, the microbial change trend was basically identical between HS and HSE group. Compared with the MC group, an increased proportion of *Odoribacter* and of *Bacteroides* was observed with HS and HSE group. On the other hand, the proportion of *Lactobacillus* genus increased significantly in the HSE group, but there was no significant increase in HS group. Compared with the MC group, the abundance of *Precotella* in HS and HSE groups decreased significantly. Finally, we inferred that HS and HSE exerted effects on constipation by affecting different genera of bacteria to influence intestinal microbiota.

**Figure 7:**
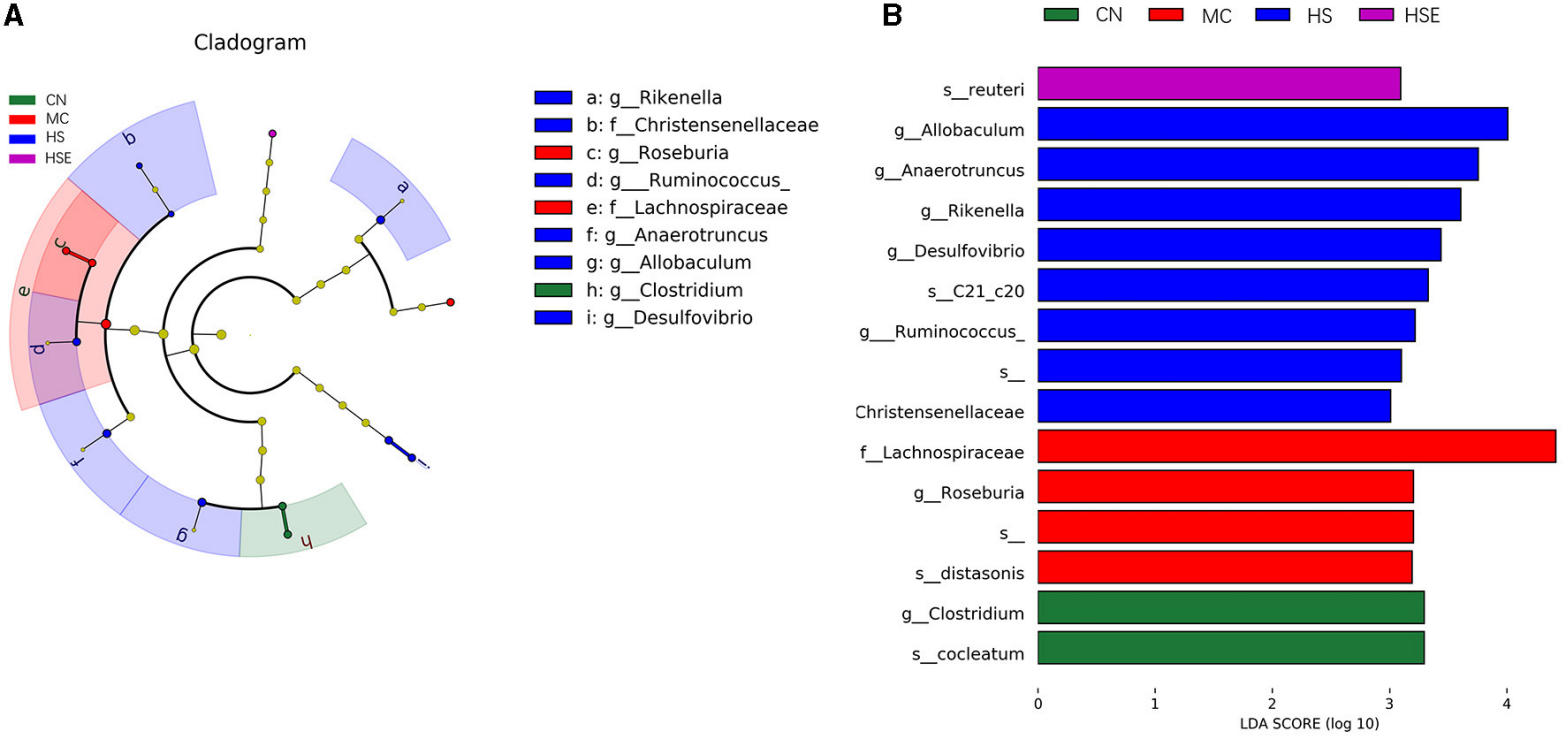
Differential classification of microorganisms in mice treated with hemp seeds for constipation. **(A)** Linear discriminant analysis effect size (LEfSe), and **(B)** Linear discriminant analysis (LDA). Data are provided as mean ± SEM (*n* = 6).

We next addressed differential changes in the relative content of microorganisms. As shown in Figure 7A, *Clostridium* was the most significantly enriched genus in the CN group, *Roseburia* and *Lachnospiraceae* were significantly enriched in the MC group, whilst *Rikenella, Ruminococcus, Anaerotruncus, Allobaculum* and *Desulfovibrio* were the significantly enriched genus in the HS group.

The results of linear discriminant analysis (LDA) provided in Figure 7B showed that *Clostridium* was the dominant genus in the NC group, *Roseburia* was the genus enriched in the MC group, and interestingly, *Allobaculum* was the genus enriched significantly in the HS group after HS treatment.

## 4 Discussion

There is a steady rise in the number of people with constipation in China with improvement of the living standards. Constipation, a functional gastrointestinal disorder that involves altered bowel movements, will seriously affect daily living and work and has become a common concern in the medical field (Rao et al., 2016). Loperamide is a drug intended for diarrhea control approved by the US Food and Drug Administration. It is often used as a model inducer for the treatment of constipation, by prolonging the duration of emptying, inhibiting peristalsis and reducing mucus in the colon. The feasibility and stability of loperamide in constipation model have been widely acknowledged (Wang et al., 2017). Fecal moisture content, time to first bowel movement, and intestinal charcoal propulsion rate are important measures used to assess gastrointestinal function (Fernández-Bañares, 2006). As one of traditional Chinese medicine with a long history in China, hemp seeds are one of the resources of affinal drug and diet, Meanwhile, hemp seeds have demonstrated potential for treating constipation, but it is still limited in popularization as a human food. This study discusses the effect of hemp seeds and water/ethanol extracts from hemp seeds in improving constipation in mice. The results showed that both hemp seeds and hemp seed extracts significantly improved the defecation parameters in mice, including fecal moisture content and time to first bowel movement. Parameters reflecting intestinal peristalsis are related to multiple aspects, such as fecal weight, fecal moisture content, time to fecal discharge, gastrointestinal transit time, small intestinal propulsion rate, and intestinal contractile activity (Gu et al., 2022). The improvement in defecation parameters indicates, to some extent, the effect of HS and HSE on intestinal peristalsis. In addition, the results of colon staining showed that HS/HSE improved to some extent the inflammatory reactions caused by constipation, which was possibly one of the reasons why hemp seeds have the effect of improving intestinal peristalsis.

We also studied the effects of hemp seeds on the serum levels of neurotransmitters and gastrointestinal hormones. MTL and GAS play an important role in regulating gastrointestinal peristalsis by stimulating the secretion of gastric acid and pepsin, promoting pyloric sphincter relaxation, gastrointestinal peristalsis and gastric emptying (Iijima et al., 2015). SP is an excitatory peptide neurotransmitter that stimulates intestinal peristalsis (Suo et al., 2014). Some studies have reported that SP stimulates interstitial cells of Cajal, induces contraction of gastrointestinal smooth muscles, and promotes gastrointestinal peristalsis (Faussone-Pellegrini, 2006). ET plays an important role in maintaining basal vascular tone and basic cardiovascular system. Constipation not only causes disorders (including intestinal obstruction and other serious diseases), but also induces or aggravates cardiovascular and cerebrovascular diseases in the elderly (Fevang et al., 2001).

In our study, after loperamide induced constipation, the levels of MTL, GAS and SP in the serum of mice in MC group were significantly lower than those in CN group, indicating that the occurrence of constipation is related to these neurotransmitters. After preventive treatment with hemp seeds and water/ethanol extracts from hemp seeds, hemp seed significantly increased the levels of MTL and GAS in serum of mice in HS group, and decreased the levels of ET, while hemp seed extract significantly increased the levels of MTL, GAS and SP in serum of mice in HSE group, and decreased the levels of ET. Extracts from hemp seeds demonstrated a stronger effect in regulating serum levels of neurotransmitters and gastrointestinal hormones. The differences observed were possibly a result of increased utility of the active ingredients in the extracts following extraction from hemp seeds. Variation in the levels of these gastrointestinal hormones would lead to a change in gastrointestinal peristalsis. We inferred that the improved intestinal propulsion rate and the shortened time to bowel movement were closely related to variation in the serum levels of such gastrointestinal hormones and neurotransmitters, suggesting that hemp seeds may alleviate the symptoms of constipation by regulating neurotransmitters and gastrointestinal hormones to stimulate intestinal peristalsis.

Disturbance of the intestinal microbiota is one of the characteristics of patients with constipation (Tian et al., 2020), It was revealed in the study by Huang et al. that patients with chronic functional constipation (CFC) had abnormalities in the number and composition of intestinal microbiota (Huang T. et al., 2018). Some studies suggested that changes in the intestinal microorganisms may alleviate or worsen constipation (Dimidi et al., 2017). In a study of pentose extracted from human milk to improve constipation in mice, it was shown that constipation was improved by increasing beneficial bacteria such as *Lactobacillus, Ruminococcaceae_UCG-014* and *Bacteroidales_S24-7* to improve the composition of intestinal microorganisms (Huang et al., 2020). In addition, hemp seeds have been widely reported to have effects on the intestinal microbiota. Changes in the intestinal microbiota in mice are primarily detected by changes in bacteria in the feces (Wang et al., 2017). Therefore, this study was of research significance by investigating whether hemp seeds could improve constipation by improving the intestinal microbiota. The results showed that after loperamide-induced constipation, significant differences in intestinal microbiota were observed between the CN and MC groups at the genus level. Our results showed that the intestinal microbiota was primarily composed of *Firmicutes and Bacteroides* and secondarily of Proteobacteria, and there was not a significant difference in the proportion of Firmicutes or Bacteroides at the genus level across the four groups, which was consistent with the study findings by (Gu et al., 2022). At the genus level, HS and HSE groups were observed with an increase in the proportion of *Odoribacter, Bacteroides* and *Lactobacillus*, and interestingly, the HS group and the HSE group were observed with a lower proportion of *Precotella* compared with the MC group. These results suggest that hemp seeds regulate the symptoms of constipation by increasing the proportion of beneficial bacteria at the genus level. *Lactobacillus* works to decompose oligosaccharides in the intestinal tract, produce organic acids (including butyric acid, acetic acid, and propionic acid), improve intestinal peristalsis, and reduce the time to bowel movement. In addition, lactobacillus produces a great amount of volatile fatty acids and inhibit the excessive production of aerobic harmful bacteria, thereby maintaining the balance of intestinal microbiota (Cao et al., 2018). It has been found that butyric acid can be used as a carbon source for intestinal microbiota to produce acetic acid (Zhuang et al., 2019).

Hemp seeds are able to regulate the intestinal microbiota, one ability of intestinal bacteria is to produce SCFAs, but this process is influenced by the number of bacteria, pH, and substrate in the intestinal tract (Liu and Zhi, 2021). SCFAs are the metabolites of dietary fiber fermented by intestinal microorganisms. There is also a strong association between SCFAs and constipation. They affect the intestinal secretion function and enhance the intestinal mucosal barrier by regulating the functions of cell subsets like enterocytes (proliferation and differentiation) and enteroendocrine cells through different mechanisms. Organic acids act on the intestinal wall to lower pH in the intestinal tract and regulate gastrointestinal peristalsis and gastrointestinal function (Malagelada et al., 2017). Acetic acid acts to up-regulate the barrier function of enterocytes of the host (Fukuda et al., 2011), whilst propionate reduces fat production, serum cholesterol levels, and carcinogenesis in other tissues (Hosseini et al., 2011). Butyrate, which is a major source of metabolic energy in the large intestine, is the most important SCFAs and help to maintain the integrity of the large intestine, control intestinal inflammation, and support the genomic stability (Jiang et al., 2020). In a study reported on hemp seed, hemp seed increased the concentration of short-chain fatty acids in intestinal contents to improve intestinal health (Jurgoński et al., 2020). SCFAs can promote the secretion of intestinal fluids, accelerate colonic motility and are even considered as candidates for the treatment of constipation (Nicholson et al., 2012). In our study, the HS group was found to have significantly higher concentrations of the six SCFAs compared with the MC group, and the HSE group was observed with higher levels of propionic acid, butyric acid and valeric acid than the MC group, but HSE (an extract from hemp seeds) appeared inferior to HS in terms of the effect of increasing the levels of SCFAs. This was possibly because that production of SCFAs is affected by the quantity of microorganisms, the ambient pH value and the substrate concentration. The amounts and proportions of SCFAs produced by different substrate vary (Ritzhaupt et al., 1998). After extraction, HSE was inferior to HS in terms of both the type and concentration of substrate, and this was possibly one of the reasons for differences in the levels of SCFAs between the HS and HSE groups.

## 5 Conclusions

This study investigated the effects of hemp seeds and hemp seed extracts in the mice with constipation. The results showed that both hemp seeds and hemp seed extracts significantly improved the moisture content of feces, shortened the time to bowel movement, and promoted intestinal peristalsis in the mice with constipation. Hemp seeds were superior to hemp seed extracts in terms of the above effects. Both affected the levels of gastrointestinal hormones in the serum, whilst hemp seed extracts exhibited a stronger effect than hemp seeds. In contrast, hemp seed extracts had less effects on the levels of SCFAs than hemp seeds. In conclusion, both hemp seeds and hemp seed extracts provide protective effects in the mice with constipation to some extent.

## Data availability statement

The datasets presented in this study can be found in online repositories. The names of the repository/repositories and accession number(s) can be found in the article/supplementary material.

## Ethics statement

The animal studies were approved by Animal Ethics Committee of Changshu Hospital Affiliated to Nanjing University of Chinese Medicine. The studies were conducted in accordance with the local legislation and institutional requirements. Written informed consent was obtained from the owners for the participation of their animals in this study.

## Author contributions

HH: Writing – original draft, Conceptualization, Formal analysis, Investigation, Methodology, Resources, Software. WY: Data curation, Formal analysis, Methodology, Visualization, Writing – original draft. DX: Conceptualization, Data curation, Methodology, Writing – review & editing. LF: Data curation, Software, Writing – original draft. GJ: Methodology, Formal analysis, Writing – review & editing. JJ: Formal analysis, Supervision, Writing – review & editing. ZF: Data curation, Formal analysis, Methodology, Writing – original draft, Writing – review & editing. JL: Funding acquisition, Investigation, Supervision, Writing – original draft, Writing – review & editing.
